# The Diagnosis of Cancer

**Published:** 1889-09-14

**Authors:** 


### Within the Wards.

### THE DIAGNOSIS OF CANCER.

To understand cancer so thoroughly as to diagnose it in its very earliest stages may enable a man to prevent untold suffering of mind and body, and to save many lives. One of the most important contributions to the improved knowledge and practical handling of the subject was made by Professor von Esmarch, of Kiel, at the Surgical Congress of Berlin, held in May of the present year. There may occur ulcerating tumours on all parts of the body, and these will often resemble cancer without being cancerous. Such tumours appear with especial frequency on the tongue and lips. What are they if they are not cancers? They are probably syphilomata, or less frequently tubercles, or actinonycotic growths. It is clear that if they are syphilomata they must not be treated as cancers, but very differently, namely, on anti-syphilitic principles. It is equally clear that if they are cancerous growths diagnosis must be prompt in order that treatment may be immediate, otherwise it will in all probability be a complete failure. How is the diagnosis between syphilomata and growths having a similar appearance on the one hand, and cancerous growths on the other, to be made at the very outset? It is to be done by the microscope, says Von Esmarch, and in most cases it can be done with certainty. "In order to procure the necessary material the surgeon should not recoil even from severe operations."

What is the significance of this to the young practitioner? It means that he must not waste time in "tinkering," such as painting with iodine and the like, when a patient consults him concerning a new growth the nature of which he does not feel absolutely certain about. He should consult with a competent and experienced operating surgeon immediately. Many general practitioners never seek the advantage of a consultation until it is suggested to them by the patient or his friends. This is a most serious error of practical judgment, and in some cases is nothing short of criminal. What is the primary and paramount duty of the doctor to his patient? To cure or relieve him in the speediest and most effectual way. In order to do this, he must be prepared not only to treat his patient according to the best of his own ability, but to place the whole of the resources of modern medical and surgical science at his disposal. That is only to be done by prompt consultation with specialists and experts in every instance where ordinary experience is insufficient. The grandest of all rules of medical ethics as between doctor and patient is for the doctor in all cases to do to the patient exactly what he would wish the patient to do to him if the positions were reversed. Is there a single doctor living who would not desire the highest skill of the most famous specialist in the world for himself in case of need? This question of the differential diagnosis of cancer is so important that it may be laid down as a general rule, that whenever a patient consults his ordinary medical attendant about a new growth which may by any possibility be of a cancerous nature, the doctor should consider it his immediate duty to seek the more experienced judgment of an operating surgeon.

